# LightGBM-Based Classification of Heart Failure Phenotypes Using Morpho-Energy Features from High-Resolution ECG

**DOI:** 10.3390/s26113397

**Published:** 2026-05-27

**Authors:** Mohamed Amin Gader, Sourour Karmani, Ridha Djemal, Carlos Valderrama Sakuyama

**Affiliations:** 1 Advanced Technologies for Medicine and Signals Laboratory (ATMS), National School of Engineering, University of Sfax, Sfax 3038, Tunisia; ridha.djemal@enis.tn; 2 Electrical Engineering Department, National School of Engineering, University of Sfax, Sfax 3038, Tunisia; 3 Department of Microelectronics & Electronics, Faculty of Polytechnic, University of Mons, 7000 Mons, Belgium; carlosalberto.valderramasakuyama@umons.ac.be; 4 Laboratory of Electronics and Microelectronics (FSM), University of Monastir, Monastir 5000, Tunisia; sourour.karmani@issatso.u-sousse.tn; 5 Higher Institute of Applied Sciences and Technology of Sousse, University of Sousse, Sousse 4000, Tunisia

**Keywords:** heart failure, HFpEF, HFmrEF, HFrEF, electrocardiogram (ECG), ECG signal processing, feature extraction, machine learning, LightGBM, cardiovascular diagnostics

## Abstract

Heart failure (HF) remains a major global health challenge, necessitating accurate yet accessible diagnostic tools. While the left ventricular ejection fraction (LVEF) is the primary metric for classifying HF into preserved (HFpEF), mid-range (HFmrEF), and reduced (HFrEF) phenotypes, conventional imaging modalities such as echocardiography are resource intensive. In contrast, the electrocardiogram (ECG) offers a low-cost, non-invasive alternative for continuous cardiac assessment. This paper proposes a multi-algorithm artificial intelligence (AI) framework for automated HF phenotype classification using high-resolution ECG signals from 303 patients with chronic heart failure from the MUSIC cohort. After preprocessing (normalization, bandpass filtering), we employed a hybrid approach combining the Pan–Tompkins algorithm for robust R-peak detection with the NeuroKit2 toolbox for the precise delineation of P, Q, S, and T waves. ECG recordings were then segmented using an adaptive beat-centric windowing strategy. From the segmented beats, we extracted a comprehensive set of temporal, morphological, and energy-based features, including RR, QRS, and QT intervals, along with P-wave, QRS-complex, and T-wave energies. These features were used to train and evaluate several ensemble machine learning models—Random Forest, XGBoost, CatBoost, LightGBM, and a stacking classifier—using a stratified 70–15–15 train–validation–test split with 5-fold cross-validation. The LightGBM model achieved the highest performance with a test accuracy of 98.45%, an AUC of 0.9989, and a macro F1-score of 0.9804, outperforming other ensembles and the stacking classifier. The results demonstrate that an AI-driven analysis of ECG-derived morpho-energy features can serve as a reliable, non-invasive screening tool for the accurate and early discrimination of HF phenotypes, potentially supporting clinical decision making and improving patient management in resource-limited settings.

## 1. Introduction

Over 64 million people worldwide suffer from heart failure (HF), which is a progressive cardiovascular disease marked by the heart’s incapacity to pump enough blood to meet the body’s metabolic demands [1,2]. Driven by population aging and the growing burden of comorbidities such as obesity, diabetes, hypertension, and coronary artery disease, its prevalence is rising [3,4]. While these comorbidities represent important risk factors for HF development, this paper focuses specifically on ECG-derived features for phenotypic classification in patients with established heart failure. Clinically, HF manifests through symptoms like dyspnea, exhaustion, and fluid retention, which have a substantial influence on patients’ quality of life and place a significant strain on healthcare resources [5]. Similarly, the analysis of other physiological signals, such as electroencephalography (EEG), has shown promise in detecting cognitive states like stress through advanced signal processing and machine learning techniques, highlighting the broader potential of AI-driven physiological signal analysis for healthcare applications [6].

The left ventricular ejection fraction (LVEF), which measures the proportion of blood expelled from the left ventricle during each cardiac cycle, is a key metric for HF diagnosis and treatment stratification. Based on the LVEF, patients are categorized into three phenotypic subtypes: heart failure with preserved ejection fraction (HFpEF, LVEF ≥ 50%), mid-range ejection fraction (HFmrEF, 41–49%), and reduced ejection fraction (HFrEF, LVEF ≤ 40%) [7]. Precise phenotypic classification is essential for guiding risk assessment, prognosis, and treatment selection.

Conventional imaging modalities for LVEF assessment include echocardiography, cardiac magnetic resonance (CMR), and nuclear imaging. Although these techniques yield precise readings, they are costly, time consuming, and reliant on specialized tools and operator expertise, which restricts their applicability for large-scale screening or ongoing monitoring.

Conversely, electrocardiography (ECG) is a widely accessible, inexpensive, and non-invasive method of recording the electrical activity of the heart [8]. Numerous studies have demonstrated that aberrant LVEF values and ventricular dysfunction are correlated with ECG parameters such as QRS duration, QT interval, T-wave morphology, and ST-segment deviations [9]. These characteristics, combined with the advent of machine learning (ML) and artificial intelligence (AI) methods, make ECG a compelling option for automated HF classification [10].

Indeed, recent developments in AI have enabled automated ECG-based HF identification with promising accuracy [11]. Broadly, existing approaches fall into two categories: traditional machine learning methods, which rely on handcrafted features such as temporal intervals, morphological descriptors, and nonlinear heart rate variability metrics [12,13], and deep learning approaches, which extract features directly from raw signals. In this paper, we deliberately focus on tree-based ensemble methods, which offer a better balance between performance, interpretability, and computational efficiency for structured tabular data, making them particularly suitable for clinical settings where model transparency is essential [14]. Despite these advancements, accurately differentiating between HF phenotypes HFpEF, HFmrEF, and HFrEF remains a clinical challenge because of their overlapping clinical manifestations [15].

To address this gap, a more robust method for HF classification may lie in combining ensemble machine learning models with a multi-domain feature extraction strategy (encompassing temporal, morphological, energy-based, and clinical characteristics) [16]. Such an approach allows models to leverage complementary information including RR intervals, QRS duration, QT interval, and waveform energy, as well as demographic and clinical data (age, sex, LVEF) to enhance predictive performance [17]. Ensemble techniques such as Random Forest, stacking, and gradient boosting (LightGBM, CatBoost, XGBoost) are particularly well suited for managing high-dimensional, heterogeneous features while guaranteeing model stability.

In this context, this paper introduces a comprehensive AI-based framework for classifying HF phenotypes from high-resolution ECG recordings of 303 patients with ischemic heart disease. There are three main contributions:

1. The design of a preprocessing and segmentation pipeline for ECG signals, featuring integrated wave detection (P, Q, R, S, T) and adaptive beat-centric windowing.
2. The extraction of a rich, multi-domain feature set comprising temporal, morphological, energy-based, and clinical descriptors to characterize cardiac dynamics.
3. A systematic evaluation and comparison of multiple ensemble ML algorithms, ultimately identifying the most accurate and stable model for distinguishing HFpEF, HFmrEF, and HFrEF subtypes.

This paper attempts to create a non-invasive, accurate, and accessible tool to enable early detection, improve risk stratification, and facilitate better clinical decision making in heart failure care by showcasing the efficacy of ECG-based AI models [18].

## 2. Related Works

The use of electrocardiographic (ECG) signals in conjunction with artificial intelligence (AI) methods for the early identification and classification of heart failure (HF) has been investigated in numerous studies [19,20]. Contemporary approaches can be broadly grouped into two paradigms: traditional machine learning models that utilize engineered features and deep learning architectures that learn representations directly from raw data.

In the domain of feature-based machine learning, Sona et al. [21] leveraged algorithms including SVM, KNN, neural networks, and decision trees to analyze circadian ECG data for predicting HF across various LVEF categories. By extracting temporal and frequency-domain variables such as QT, QRS, and ST intervals from 24-h recordings segmented into one-hour windows, they achieved an accuracy exceeding 91% using a tree-based classifier. Similarly, Alkhodari et al. [22] distinguished between HF subtypes (HFpEF, HFmrEF, and HFrEF) using clinical and demographic factors alone, such as age, BMI, diabetes, and arrhythmia history. Their method, which combined CNN-based feature extraction with machine learning classifiers, achieved an accuracy of 90.1%, thereby demonstrating that clinical markers can significantly aid in HF prediction even without temporal ECG segmentation. In a hybrid vein, Botros et al. [23] investigated the combination of CNN-derived representations with SVM-based classification, reporting encouraging outcomes in distinguishing HF patients from healthy controls. Our previous research focused on deep learning-based arrhythmia detection from ECG signals [24,25]. In contrast, this paper investigates ensemble machine learning approaches for heart failure phenotyping, demonstrating the versatility of ECG analysis across different cardiac pathologies.

Conversely, deep learning techniques have proven effective at automatically learning spatiotemporal representations from raw ECG data. For instance, Zhang et al. [26] presented a CNN-LSTM architecture using 30-min ECG recordings divided into 12-s intervals, capturing both morphological aspects and long-term temporal dependencies to reach an accuracy of 99.09% on the MIMIC-III and BIDMC datasets. Separately, Kwon et al. [27] developed a CNN-based model to identify HFpEF from brief (10-s) ECG segments augmented with demographic data. Trained on a multi-institutional cohort of over 34,000 patients, their approach demonstrated significant robustness and generalization. Further underscoring the value of AI-enhanced ECG interpretation, Akbilgic et al. [28] introduced ECG-AI, which is a deep learning framework integrating multi-cohort datasets for HF prediction and early risk stratification.

In summary, prior research illustrates two dominant trends: (1) machine learning models dependent on hand-engineered ECG and clinical parameters and (2) deep learning architectures capable of directly learning discriminative patterns from raw data. While these contributions are significant, several limitations persist in the literature. While deep learning approaches have demonstrated excellent performance, they typically require large datasets, and their “black-box” nature limits clinical adoption. Our work positions itself as a pragmatic alternative: leveraging interpretable ECG features with high-performing yet transparent ensemble models.

Many studies focus either on fully automated deep models or manually crafted descriptors with inadequate integration of multi-domain data that combines temporal, morphological, and clinical markers. For instance, Sona et al. [21] relied exclusively on temporal and frequency–domain ECG features (TP, QT, QRS, ST intervals, entropy, instantaneous frequency) without integrating morphological energy descriptors or clinical markers, achieving 91.2% accuracy. Conversely, Alkhodari et al. [22] utilized only clinical profiles (age, BMI, diabetes, arrhythmia history), omitting the rich electrical information contained in ECG waveforms, and attained 90.1% accuracy. Kwon et al. [27] combined limited ECG features (QRS, P-wave, T-wave) with demographic data but did not incorporate energy-based morphological parameters that reflect ventricular contraction strength, resulting in 86.6% accuracy. In contrast, our approach integrates temporal intervals (RR, QRS, QT), morphological energies (P-wave, QRS, T-wave), and clinical context, achieving 98.45% accuracy—representing a 7–12% absolute improvement over these single-domain methods. Moreover, numerous methods employ fixed-length signal segments, which may fail to capture patient-specific rhythm variability, and many rely on single-model designs without fully exploiting the advantages of ensemble strategies. These gaps collectively highlight the need for a more comprehensive framework that seamlessly integrates robust classification, rich multi-domain feature representations, and adaptive, patient-centric segmentation. Building upon these insights, the following section presents a methodology designed to incorporate these components into a coherent workflow for reliable HF phenotyping.

While deep learning models such as CNNs and CNN-LSTM architectures [26,27] learn representations directly from raw waveforms—requiring large cohorts (often >10,000 patients) for effective training and generalization—our feature-based ensemble strategy leverages clinically interpretable ECG parameters (RR, QRS, QT intervals; P/QRS/T-wave energies). This distinction is critical for two reasons. First, the moderate size of the MUSIC cohort (n = 303) favors handcrafted feature approaches over representation learning, which is prone to overfitting in small datasets. Second, the features we extract maintain direct physiological meaning (e.g., QRS prolongation indicating conduction delay, reduced QRS energy reflecting impaired ventricular contractility), whereas CNN-derived features operate as “black-box” representations that are difficult to interpret in clinical settings. Consequently, our approach achieves state-of-the-art performance (98.45% accuracy) while preserving the transparency required for clinical decision support—a balance that deep learning methods trained on similarly sized cohorts have not demonstrated.

## 3. Proposed Methodology

The overall pipeline for heart failure classification is organized into six sequential stages:

1. Data acquisition: collection of high-resolution ECG recordings and associated clinical metadata.
2. Preprocessing: signal normalization and robust detection of fiducial points (P, Q, R, S, T waves).
3. Segmentation: application of an adaptive, beat-centric windowing strategy centered on R-peaks.
4. Feature extraction: computation of temporal, morphological, energy-based, and clinical parameters from segmented beats.
5. Classification: training and evaluation of ensemble machine learning models (CatBoost, LightGBM, XGBoost, Random Forest, and a stacking ensemble).
6. Performance evaluation: comprehensive assessment using accuracy, recall, F1-score, confusion matrices, and ROC curves.

**Figure 1:**
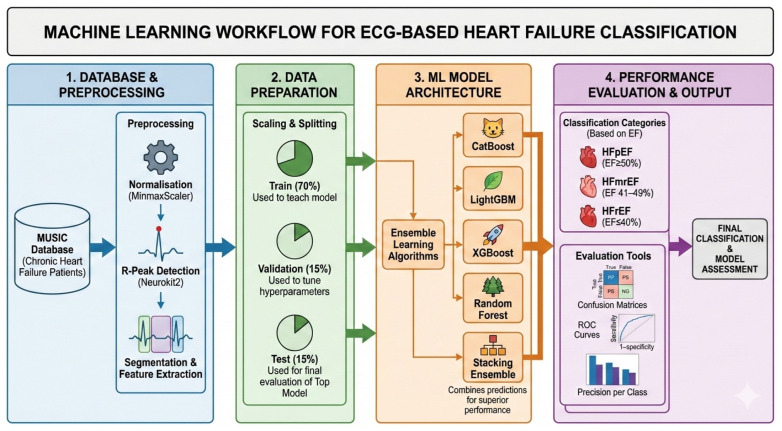
Overall architecture of the proposed heart failure classification framework: six-step machine learning pipeline: (1) data acquisition, (2) preprocessing, (3) segmentation, (4) feature extraction, (5) classification using ensemble models, and (6) performance evaluation. The workflow integrates adaptive ECG analysis with clinical metadata to predict heart failure phenotypes.

A schematic of the general structure is shown in Figure 1. Our approach aims to provide a more comprehensive and reliable depiction of cardiac electrical activity by fusing high-resolution ECG signals with clinical data. The synergy between adaptive segmentation, multi-domain feature extraction, and ensemble learning is designed to ensure the stable and precise prediction of HF phenotypes.

Consequently, this pipeline supports early risk assessment and clinical decision making by delivering an effective and interpretable tool to differentiate between HFpEF, HFmrEF, and HFrEF.

The extracted feature matrix is provided simultaneously as input to all five classifiers (CatBoost, LightGBM, XGBoost, Random Forest, and the stacking ensemble). Each model is trained independently on the same training data using 5-fold cross-validation. Final predictions are obtained from the best-performing individual model (LightGBM) or through the stacking meta-learner.

### 3.1. Dataset and Patients’ Enrollment

This paper utilized the publicly available MUSIC (Sudden Death in Heart Failure) database, which is hosted on PhysioNet [29]. The database provides high-resolution resting ECG recordings sampled at 1000 Hz with a three-lead orthogonal layout (X, Y, Z), which is accompanied by comprehensive clinical and demographic metadata. For all subsequent analyses, we utilized Lead X exclusively. Lead X corresponds to the left–right horizontal axis in the orthogonal Frank lead system and provides an optimal visualization of QRS complex morphology and T-wave characteristics—electrophysiological features central to HF phenotype discrimination. This single-lead approach also enhances translational potential to wearable and mobile ECG platforms, which typically employ 1–2 lead configurations.

The cohort consisted of 303 patients with chronic heart failure, aged 18 to 89 years, enrolled in the MUSIC study, which is a prospective registry designed to evaluate risk factors for sudden cardiac death in heart failure patients. All participants underwent standardized clinical examination and echocardiography to measure the left ventricular ejection fraction (LVEF). In accordance with ASE/EACVI guidelines [30], patients were stratified into three heart failure phenotypes (see LVEF on Table 1): HFpEF (n = 63, 20.8%), HFmrEF (n = 54, 17.8%), and HFrEF (n = 186, 61.4%). All LVEF measurements were performed by certified sonographers following institutional protocols. The study protocol was approved by the relevant institutional review boards and complied with the Declaration of Helsinki; written informed consent was obtained from all participants. Inclusion criteria required a diagnosis of stable ischemic cardiomyopathy and the availability of standardized echocardiographic data. Key exclusion criteria comprised congenital heart disease, acute decompensated heart failure at the time of recording, persistent atrial fibrillation, implanted cardiac devices (e.g., pacemakers or ICDs), or severe systemic illnesses (e.g., advanced hepatic, renal, or malignant diseases).

The collected clinical data included age, sex, body mass index (BMI), smoking status, diabetes, hypertension, and a history of myocardial infarction (MI). Patients with incomplete clinical data (demographics, medications, comorbidities) were excluded from the study. However, for ECG-derived features, occasional missing values occurred after segmentation (e.g., due to noisy segments where wave delineation failed). These missing values were handled using K-nearest neighbors (KNN) imputation during the preprocessing pipeline.

The dataset was split at the patient level into training (70%), validation (15%), and test (15%) sets while maintaining the distribution of HF phenotypes. Critically, all segments originating from a single patient were assigned to the same data split (training, validation, or test) to prevent data leakage. Segmentation and feature extraction were performed after the patient-level split to ensure that no information from test patients influenced the training process.

Table 1 summarizes the key demographic and clinical characteristics of the cohort.

### 3.2. Preprocessing and Feature Extraction

Given the susceptibility of ECG signals to inter-patient variability, baseline drift, and noise, a rigorous preprocessing pipeline was implemented to ensure reliable feature extraction and classification. The processing sequence is (1) bandpass filtering, (2) amplitude normalization, and (3) fiducial point detection.

#### 3.2.1. Bandpass Filtering

A dual-stage bandpass filter (0.5–40 Hz) was first applied to suppress baseline wander and high-frequency noise. This can be represented as:

- High-pass filter (0.5 Hz): suppresses baseline wander, which is mainly caused by respiratory movements.
- Low-pass filter (40 Hz): reduces high-frequency noise, such as muscle activity or electrical interference.

Mathematically, this corresponds to a bandpass filter:

$$y\left(t\right)=x\left(t\right)*h\left(t\right),\;H\left(f\right)=\left\{\begin{array}{cc} 1 & \mathrm{if}\;0.5\leq f\leq 40\;\mathrm{Hz}\\ 0 & \mathrm{otherwise} \end{array}\right.$$

where x(t) is the raw ECG signal, h(t) is the filter response, and y(t) is the filtered signal.

#### 3.2.2. Normalization

Following bandpass filtering, all ECG signals were normalized to the range [0, 1] using MinMaxScaler, which is a standard normalization technique that reduces amplitude variability across patients and stabilizes machine learning model training [31]. This step ensures that extracted morphological features are comparable across recordings irrespective of acquisition settings.

#### 3.2.3. R-Peak and Fiducial Points Detection

On the filtered and normalized signals, the accurate automatic detection of ECG waves (P, QRS, and T) is critical for deriving reliable features. To this end, we employed a hybrid approach that integrates the robustness of the Pan–Tompkins algorithm for R-peak detection with the sophisticated delineation capabilities of the NeuroKit2 toolbox [32] for identifying the remaining fiducial points (P, Q, S, and T). The detection pipeline comprised three main steps:

1. R-peak detection (Pan–Tompkins): The Pan–Tompkins algorithm was applied, involving differentiation, squaring, and moving-window integration:
  $$y^{\prime }\left(t\right)=x\left(t\right)-x\left(t-1\right)\;\left(\mathrm{derivative}\right)$$
  $$y^{2}\left(t\right)={\left(y^{\prime }\left(t\right)\right)}^{2}\;\left(\mathrm{squaring}\right)$$
  $$y_{\mathrm{int}}\left(t\right)=\frac{1}{N}\sum_{i=0}^{N-1}y^{2}\left(t-i\right)\;\left(\mathrm{integration}\right)$$
  Peaks are then identified by comparing $y_{\mathrm{int}}\left(t\right)$ against an adaptive threshold.
2. Wave delineation (P, Q, S, T): Two complementary methods were used:
  - Primary method (peak search): Local maxima/minima were identified within predefined windows around each R-peak (e.g., Q wave in [R−40ms,R], and the T wave in [R+60ms,R+400ms].)
  - Fallback method (Discrete Wavelet Transform, DWT [33]): The ECG was decomposed into wavelet coefficients $W_{j,k}$ to isolate frequency components associated with P, QRS, and T waves:
    $$W_{j,k}=\sum_{n}x\left(n\right)\;\psi_{j,k}\left(n\right)$$
    where $\psi_{j,k}$ is a wavelet function dilated by *j* and translated by *k*.
3. Noise Filtering: Invalid fiducial points (e.g., NaN values or physiologically implausible intervals) were removed to ensure consistency in the extracted features.

**Figure 2:**
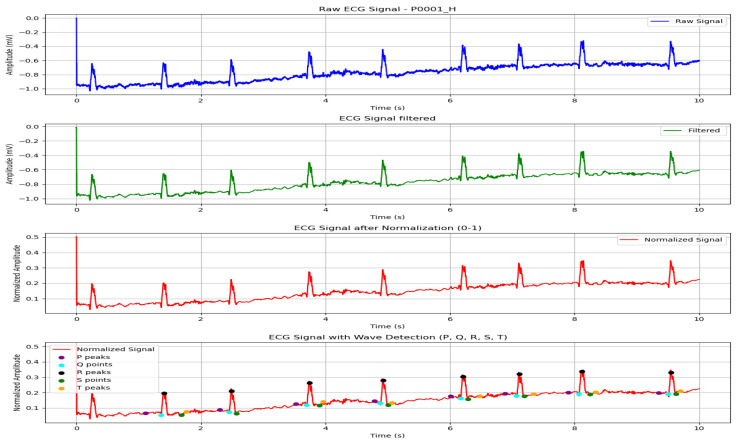
Example of a 10-s ECG segment for patient P0001_H. The panels show (**top**) the raw signal, (**middle**) the filtered (**green**) and normalized (**red**) signal, and (**bottom**) the results of R-peak detection and P-QRS-T wave delineation.

This hybrid methodology ensures precise fiducial point identification while maintaining robustness against noise and inter-patient variability. Figure 2 illustrates a 10-s ECG segment from patient P0001_H, showing how red filtering, normalization, and the hybrid detection pipeline accurately identify R-peaks and P–QRS–T waves.

#### 3.2.4. Segmentation

Following preprocessing, ECG signals were segmented using an adaptive, beat-centric approach to preserve physiologically consistent cardiac dynamics and augment the effective number of training samples. The segmentation workflow involved the following:

1. R-peak detection: R-peaks were identified, defining consecutive RR intervals ($RR_{i}$).
2. Average cardiac cycle computation: The mean R-R interval was calculated as
  $$RR_{\mathrm{avg}}=\frac{1}{N}\sum_{i=1}^{N}RR_{i}$$
  where *N* is the total number of detected beats.
3. Window size determination: Each segment was designed to encompass approximately $N_{b}=60$ beats with the number of samples per window given by
  $$W=RR_{\mathrm{avg}}\times f_{s}\times N_{b}$$
  where $f_{s}=1000$ Hz is the ECG sampling rate.
4. Sliding window with overlap: Adjacent windows were overlapped by 50% to ensure continuity, resulting in a stride length
  $$P=\frac{W}{2}$$
5. Number of segments: For an ECG recording of length *L* samples, the total number of segments was
  $$N_{\mathrm{seg}}=\left\lfloor \frac{L-W}{P}\right\rfloor +1$$

Illustrative example: For a recording with L=180 beats, using $N_{b}=60$ beats per window and 50% overlap (P=30), the resulting number of segments is

$$N_{\mathrm{seg}}=\frac{180-60}{30}+1=5.$$

For patient P0001_H, segmentation yielded five windows of roughly 70 s each, with 50% overlap, producing 37 effective segments covering 22 min of ECG ($RR_{\mathrm{avg}}=1163.1$ ms).

**Figure 3:**
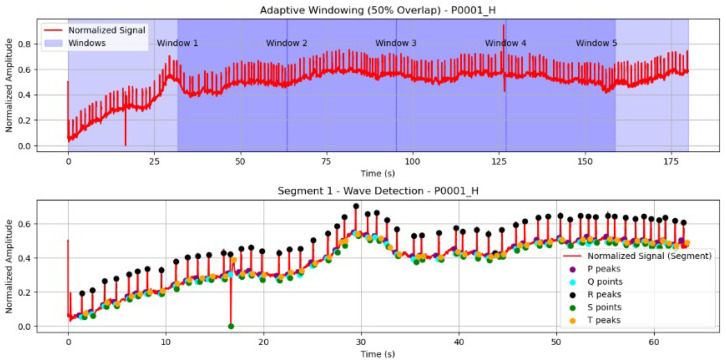
Beat-centric segmentation strategy: (**Top**) A 180-s ECG recording from patient P0001_H divided into five overlapping windows (50% overlap, color-coded). Each window encompasses approximately 60 cardiac cycles to ensure consistent morphological context. (**Bottom**) Magnified view of a single 60-beat segment showing R-peak detection (red markers) and P-QRS-T wave delineation (blue annotations). This segmentation approach preserves beat-to-beat variability while augmenting the effective number of training samples.

This adaptive segmentation offers three key advantages: (i) it increases the number of training samples; (ii) it preserves local heart rhythm variations (e.g., arrhythmias); and (iii) controlled redundancy from overlapping segments enhances model robustness. This strategy is consistent with earlier deep learning research [34], which showed that clustering several cardiac cycles per segment stabilizes morphological, energy and temporal characteristics. Figure 3 illustrates how the 180-s ECG recording is divided into overlapping windows, highlighting the preservation of beat-to-beat variability and the creation of multiple effective segments for model training.

#### 3.2.5. Feature Extraction

From each segmented beat, a set of attributes was calculated to capture the ECG’s morphological, energy, and temporal characteristics. First, key temporal intervals were extracted as reliable markers of cardiac electrophysiology and HF risk predictors [20]: the mean R-R interval (RR_mean), mean QRS duration (QRS_mean), and mean QT interval (QT_mean).

Additionally, the energy of the P-wave, QRS complex, and T-wave components was computed. These energy-based features provide complementary information on atrial depolarization, ventricular depolarization, and ventricular repolarization, respectively, and they have been shown to improve classification performance in automated ECG analysis.

In summary, the combination of interval-based and energy-based features enhances the diagnostic framework’s robustness by integrating both rhythm-related and morphology-related markers. The complete list and descriptions of these extracted features are detailed in Table 2.

Quality-Controlled Feature Aggregation: Features were computed exclusively from beats that passed quality control criteria. Invalid fiducial points (None or NaN values) were automatically filtered out during wave delineation. For each segmented window, temporal intervals and energy features were computed as the median across all valid beats, providing robustness against occasional detection errors and outlier beats. After aggregation, any remaining missing feature values at the segment level were imputed using K-nearest neighbors (KNN, k=5) during the preprocessing pipeline. The missing value rates were: QRS_duration (29.2%), QT_interval (20.8%), P_Energy (4.8%), QRS_Energy (3.7%), and T_Energy (0.02%). The higher rates for QRS and QT reflect the challenge of automated wave delineation in patients with severe heart failure. KNN imputation preserves the underlying data structure while retaining clinically informative cases that would otherwise be excluded.

## 4. Machine Learning Methodologies and Training Configurations

In this paper, we employed four ensemble-based machine learning models to categorize patients into HFpEF, HFmrEF, and HFrEF groups: CatBoost, LightGBM, XGBoost, and Random Forest. These models were selected for their proven performance in handling structured tabular data and their ability to capture complex nonlinear relationships.

The primary evaluation metric was classification accuracy. To optimize model performance, hyperparameters were tuned systematically using GridSearchCV with 5-fold cross-validation. A predefined hyperparameter grid was explored for each model, encompassing learning rates, the number of estimators, tree depths, and other model-specific parameters. For CatBoost, early stopping based on a validation set was implemented to prevent overfitting.

Our preprocessing pipeline included K-nearest neighbors (KNN) imputation for missing values, the standardization of features using StandardScaler, and label encoding for the target classes. To ensure robust evaluation, the dataset was split into training (70%), validation (15%), and test (15%) sets. Finally, a stacking ensemble was constructed using the individually optimized base models with a multinomial logistic regression acting as the meta-classifier to further enhance classification robustness.

This comprehensive approach ensures that each model is carefully tuned and validated, leveraging the complementary strengths of multiple ensemble methods for reliable heart failure classification.

Hyperparameter Optimization Protocol: For each model, we conducted systematic grid search using 5-fold stratified cross-validation on the training partition (70% of patients). The optimization objective was macro-averaged F1-score to account for class imbalance (HFrEF: 61.4%, HFmrEF: 17.8%, HFpEF: 20.8%). Table 3 summarizes the search space and selected configurations. For boosting models (CatBoost, LightGBM, XGBoost), we employed early stopping with a patience of 50 rounds on a 10% validation split within each cross-validation fold to prevent overfitting. The optimal hyperparameters were selected based on the highest mean validation F1-score across folds with the constraint that the training–validation performance gap remained below 5% to ensure generalization.

### 4.1. CatBoost

CatBoost is a gradient boosting technique specifically optimized for categorical data and resistant to overfitting [35]. Tuned hyperparameters included the number of iterations [500, 1000], learning rate [0.01, 0.1], depth [6, 8, 10], and early stopping rounds [50, 100]. We determined an optimal configuration: iterations = 1000, learning rate = 0.01, depth = 8, early stopping rounds = 50.

### 4.2. LightGBM

LightGBM is a histogram-based gradient boosting framework designed for high efficiency with large datasets [36]. We tuned the hyperparameters to the following values: n_estimators [500, 1000], learning rate [0.01, 0.1], max_depth [6, 8, 10], and num_leaves [15, 30]. We found an optimal configuration: n_estimators = 1000, learning rate = 0.01, max_depth = 8, num_leaves = 30.

### 4.3. XGBoost

XGBoost is a widely used regularized gradient boosting algorithm. We tuned the hyperparameters to the following values: n_estimators [500, 1000], learning rate [0.01, 0.1], and max_depth [6, 8, 10]. Afterwards, we found the optimal values: n_estimators = 1000, learning rate = 0.01, max_depth = 8.

### 4.4. Random Forest

Random Forest is an ensemble method that builds multiple decision trees and aggregates their predictions. We tuned the hyperparameters to the following values: n_estimators [100, 200, 300] and max_depth [5, 10, 20]. Then, we found an optimal setting: n_estimators = 200, max_depth = 10.

### 4.5. Stacking Ensemble

A stacking classifier was implemented by combining the individually optimized CatBoost, LightGBM, XGBoost, and Random Forest models. Logistic regression was used as the meta-estimator. No additional hyperparameter tuning was performed on the stacking classifier itself.

### 4.6. Summary of Hyperparameters

For clarity, Table 3 summarizes the tuned hyperparameter ranges and the selected optimal configuration for each model.

## 5. Results

All analyses were performed on a workstation equipped with an Intel Core i5-13420H processor and 24 GB of RAM.

### 5.1. ECG Feature Summary and Statistical Analysis

Table 4 summarizes the extracted ECG characteristics, including the RR interval, QRS duration, QT interval, P-wave energy, QRS energy, and T-wave energy for each of the three heart failure subtypes (HFpEF, HFmrEF, and HFrEF). Values are presented as median (interquartile range, Q1–Q3) due to the non-normal distribution of ECG features confirmed by Shapiro–Wilk testing.

Given that ECG data typically follow a non-normal distribution, normality was first assessed using the Shapiro–Wilk test. Since the results confirmed deviations from normality, group comparisons were performed using the non-parametric Kruskal–Wallis test. Subsequently, post hoc pairwise comparisons were conducted with Mann–Whitney U tests to identify significant differences between specific HF subtypes.To account for multiple comparisons, we applied a Bonferroni correction, adjusting the significance threshold to *p* < 0.017 (0.05/3). Furthermore, nonlinear features, such as entropy-based measures [37], can be applied to ECG signals to capture subtle dynamics and complex irregularities that are not fully characterized by conventional temporal descriptors (e.g., RR, QRS, and QT intervals). The wide interquartile ranges observed in some features reflect the substantial clinical heterogeneity inherent in heart failure populations.

### 5.2. Correlation Analysis of ECG Features

**Figure 4:**
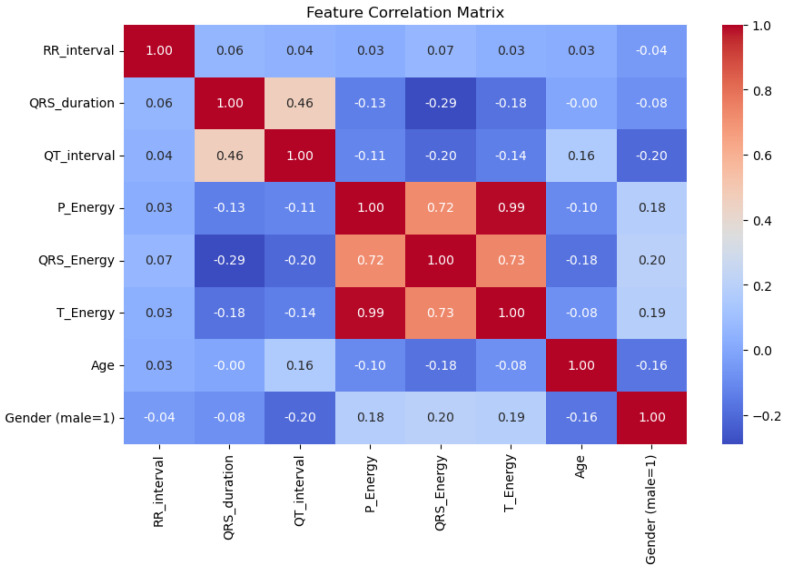
Spearman correlation matrix with values from −1 to 1. Correlation heatmap for ECG features and demographic variables. Strong positive correlations are represented in red, and weak correlations are represented in blue.

To examine monotonic relationships between ECG characteristics and demographic variables, we performed Spearman’s rank correlation analysis, which is more appropriate for non-normally distributed data. The resulting correlation heatmap is displayed in Figure 4, where strong positive correlations are represented in darker red and weak correlations are reported in blue.

### 5.3. Model Performance and Evaluation

Each model’s performance was evaluated using validation accuracy, test accuracy, AUC, macro F1-score, and training time. Table 5 presents a comprehensive comparison of the classifiers considered in this paper: CatBoost, LightGBM, XGBoost, Random Forest, and the stacking ensemble.

The results confirm the efficacy of ECG-derived features in distinguishing between HFpEF, HFmrEF, and HFrEF with all classifiers achieving test accuracies above 95%. Future improvements could address class imbalance through synthetic signal generation techniques, as demonstrated in EEG studies [38].

Notably, LightGBM achieved the highest performance with a validation accuracy of 98.66%, a test accuracy of 98.45%, and the best AUC score (0.9989). It also exhibited the lowest training time among the boosting-based models (2.88 s), highlighting its computational efficiency.

In terms of per-class performance, LightGBM achieved F1-scores of 0.962 (HFpEF), 0.967 (HFmrEF), and 0.993 (HFrEF), demonstrating strong minority class performance despite the class imbalance.

Following closely, XGBoost and CatBoost achieved test accuracies of 97.68% and 97.26%, respectively, both with high AUC values (0.9973 and 0.9965). While Random Forest maintained competitive performance (test accuracy 95.77%, AUC 0.9963) and the fastest training time, it demonstrated reduced precision compared to boosting methods.

The stacking ensemble delivered strong performance (test accuracy 98.19%, AUC 0.9979) and the lowest cross-validation variability, indicating high stability. However, this came at the expense of a substantially longer training time (121.37 s).

**Figure 5:**
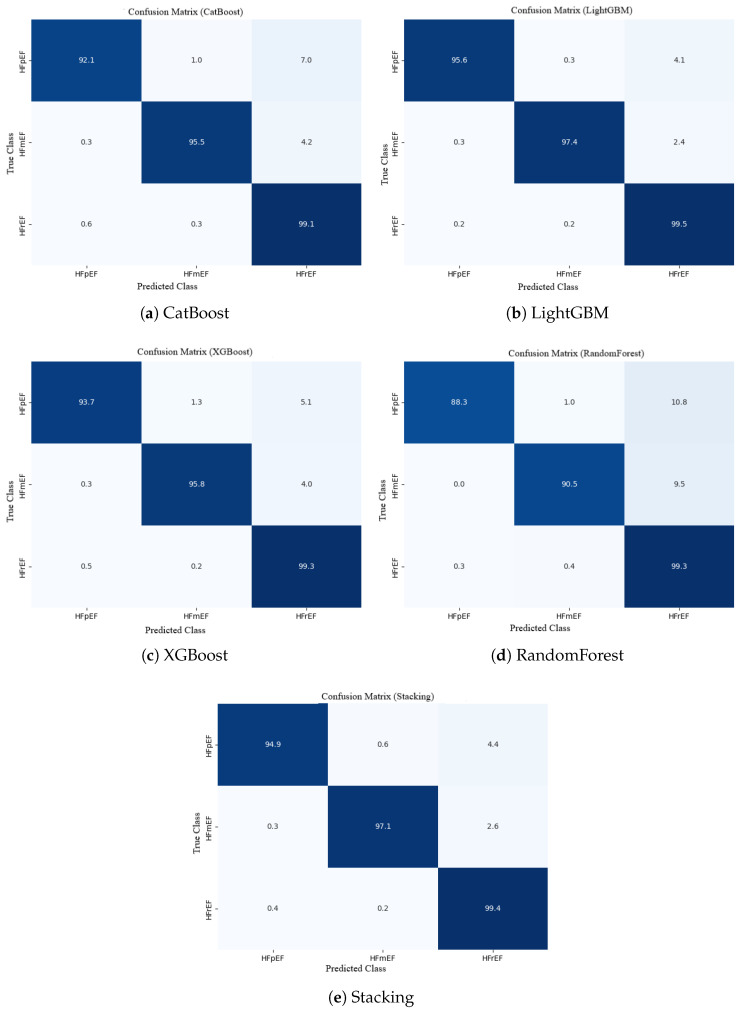
Confusion matrices (in %) for the classification models on the test set. High diagonal values indicate accurate predictions with low off-diagonal errors across all HF categories.

Figure 5 presents the normalized confusion matrices for all five models, illustrating classification performance across the three HF categories. All models show strong diagonal dominance, reflecting low misclassification rates. Specifically, LightGBM achieved the highest per-class accuracies: 95.6% for HFpEF, 97.4% for HFmrEF, and 99.5% for HFrEF.

**Figure 6:**
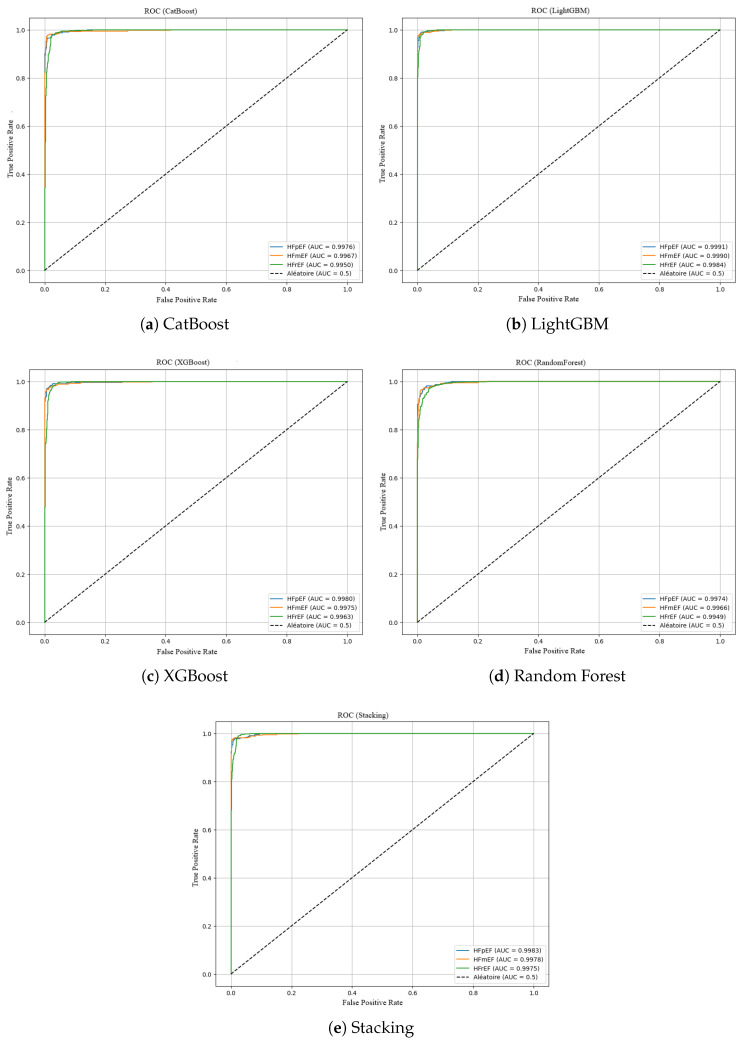
ROC curves for the classification models on the test set, showing class-specific performance for HFpEF, HFmrEF, and HFrEF. High AUC values (close to 1.0) indicate strong discriminatory power across all categories.

Furthermore, the ROC curves for each model are displayed in Figure 6, demonstrating strong discriminatory power across all HF subtypes. LightGBM again achieved the highest class-specific AUC values (0.9991 for HFpEF, 0.9990 for HFmrEF, and 0.9984 for HFrEF).

### 5.4. Model Stability and Overall Comparison

**Figure 7:**
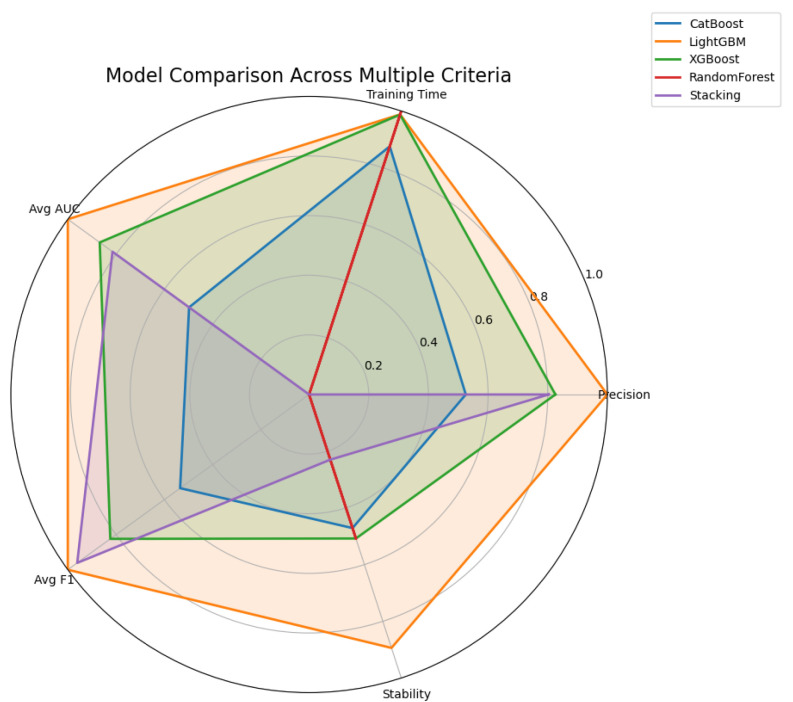
Radar chart comparing models across multiple performance criteria. LightGBM dominates across most metrics, particularly in Precision, Avg AUC, and Stability, making it the most well-rounded and robust model.

**Figure 8:**
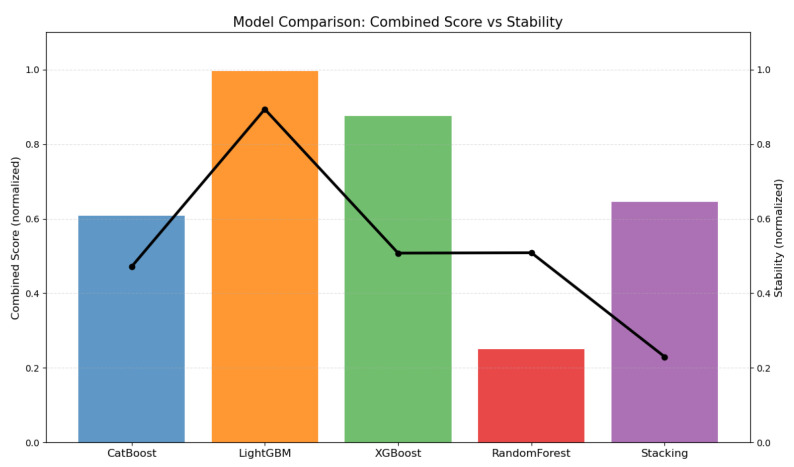
Comparison of models based on the combined score and stability. LightGBM achieves the highest combined score (0.9970) and stability (0.8938), reflecting its superior balance of performance and reliability.

To provide a holistic comparison, Figure 7 and Figure 8 integrate multiple performance and stability metrics. The radar chart (Figure 7) compares models across key criteria: Precision, Avg AUC, Avg F1, Stability, and Training Time. LightGBM dominates across most dimensions, particularly in Precision, Avg AUC, and Stability.

Complementing this, the bar plot (Figure 8) compares models based on a combined score and stability metric. LightGBM achieves the highest combined score (0.9970) and stability (0.8938), reflecting its superior balance of performance and reliability.

In summary, the comparative analysis demonstrates that LightGBM provides the best trade-off between predictive accuracy, stability, and computational efficiency, making it the most suitable model for scalable and reliable HF phenotype classification in clinical settings.

## 6. Discussion

Our comparative analysis highlights the diversity of methods, input features, and segmentation techniques employed in recent research on heart failure (HF) classification, as summarized in Table 6. To achieve optimal classification accuracy across HF subtypes (HFpEF, HFmrEF, and HFrEF), different methodologies leverage various information sources, ranging from clinical indicators to raw ECG waveforms.

Sona et al. [21] examined ECG-derived temporal and frequency-domain features including TP, QT, QRS, and ST intervals, mean entropy, and mean instantaneous frequency from 24-h recordings segmented into 1-h intervals. Their best model, a tree-based classifier, reached an accuracy of 91.2%. While this approach demonstrated the predictive value of ECG features, its coarse 1-h segmentation may limit its ability to capture brief electrophysiological fluctuations occurring over shorter timescales.

In contrast, Zhang et al. [26] proposed a CNN-LSTM framework that combined spatial and temporal features extracted from 30-min ECG recordings divided into 12-s intervals. By employing fine-grained segmentation, they achieved an impressive accuracy of 99.09% on the MIMIC-III and BIDMC datasets. However, their heavy reliance on a complex deep-learning architecture may pose challenges for clinical interpretability and deployment in resource-constrained settings.

Alkhodari et al. [22] adopted a different strategy, stratifying patients based solely on clinical markers such as age, BMI, diabetic status, arrhythmic history, and prior myocardial infarction. Their CNN-based model achieved an accuracy of 90.10%, underscoring the utility of non-ECG data. Nevertheless, relying exclusively on clinical profiles omits the rich electrical information contained in the ECG, which could restrict the model’s generalizability to populations with different clinical characteristics.

Kwon et al. [27] evaluated a large multi-hospital cohort (n = 34,103) using brief 10-s ECG segments augmented with demographic data. Their CNN model attained an accuracy of 86.6%, demonstrating the feasibility of large-scale HF screening. Yet, its relatively lower performance compared to feature-rich, ECG-based models suggests that integrating temporal ECG descriptors with demographic data could yield improved outcomes.

In this paper, we have shown that optimized ensemble machine learning models, applied to carefully curated ECG-derived features (RR, QT, and QRS intervals, along with P-wave, T-wave, and QRS complex energies), achieve state-of-the-art performance. As summarized in Table 6, using 1-min adaptive segments from the MUSIC database (303 patients), our models obtained test accuracies of 97.26% (CatBoost), 98.45% (LightGBM), 97.68% (XGBoost), 95.77% (Random Forest), and 98.19% (Stacking). All comparative accuracy values discussed in this section are test-set metrics reported in the respective original studies. These results demonstrate high internal performance on the MUSIC dataset, comparing favorably with earlier ECG-only [21,26] and clinical-profile-based approaches [22]. However, we acknowledge that external validation on independent cohorts is necessary to confirm robustness and generalizability.

Importantly, our findings emphasize the clinical relevance of temporal ECG metrics such as QT and QRS intervals which reflect underlying electromechanical cardiac dynamics. The exceptional accuracy, stability, and computational efficiency of LightGBM highlight its strong potential for integration into practical clinical decision-support systems that require robustness, speed, and interpretability. The MUSIC cohort exhibits moderate class imbalance (HFrEF: 61.4%, HFpEF: 20.8%, HFmrEF: 17.8%). Notably, our LightGBM model achieved excellent per-class performance despite this imbalance with sensitivities of 99.5%, 95.6%, and 97.4% for HFrEF, HFpEF, and HFmrEF, respectively. Given the already high baseline performance (98.45% test accuracy), the expected absolute improvement from explicit class balancing is limited—likely below 1% in overall accuracy. The primary benefit would instead be improved stability and probability calibration for minority classes. We also caution that synthetic oversampling in the feature space may generate physiologically implausible combinations of ECG parameters, potentially degrading external validity. A more principled approach would involve the targeted collection of additional minority class samples in prospective studies.

In summary, this paper advances the field of ECG-based HF phenotyping: (1) validating the high predictive power of a focused set of morpho-energy ECG features, (2) demonstrating the superiority of LightGBM among ensemble models, and (3) proposing a scalable, efficient pipeline that bridges advanced computational techniques with clinical applicability particularly in settings where access to echocardiography or specialist expertise is limited. Although deep learning models (CNNs, LSTMs) have shown promising results in the literature [26], our methodological choice is motivated by several factors. First, the moderate size of our cohort (n = 303) favors handcrafted feature approaches over representation learning. Second, interpretability is crucial for clinical adoption; ensemble models enable the identification of the most discriminative ECG features (via SHAP or feature importance analysis). Third, the computational efficiency of LightGBM (2.88 s) makes it deployable in resource-limited settings. These considerations justify our feature-based approach as a pragmatic and clinically relevant solution.

## 7. Conclusions

This paper demonstrates that a carefully selected set of ECG-derived features, processed through an optimized machine learning pipeline, can effectively classify heart failure patients into preserved (HFpEF), mid-range (HFmrEF), and reduced (HFrEF) ejection fraction phenotypes. The proposed framework serves as an efficient and supplementary tool to routine echocardiography, addressing the need for accessible and non-invasive screening methods.

By leveraging advanced ensemble models, particularly gradient boosting techniques such as LightGBM, our approach achieves superior accuracy, stability, and computational efficiency compared to earlier ECG-only and clinical-profile-based methods. Specifically, LightGBM attained a test accuracy of 98.45%, an AUC of 0.9989, and a macro F1-score of 0.9804, outperforming other ensemble models and the stacking classifier while maintaining a low training time.

Our results underscore the clinical relevance of temporal ECG metrics including QT and QRS intervals, which reflect underlying electrical and mechanical cardiac dynamics. The use of adaptive 1-min segmentation from the MUSIC dataset enabled the capture of essential temporal patterns while preserving computational feasibility.

Importantly, this methodology holds significant potential to simplify HF assessment in resource-limited environments where access to echocardiography or specialized expertise is constrained. The framework provides a cost-effective, efficient, and interpretable solution for continuous cardiac monitoring, early HF detection, and treatment response evaluation.

The primary limitation of this paper is the absence of external validation. Future work must prioritize prospective validation on independent, multi-center cohorts to confirm generalizability. Additionally, the integration of deep learning architectures and multi-lead ECG analysis could further enhance predictive performance. Furthermore, a complete end-to-end timing analysis of the full pipeline—including preprocessing, segmentation, feature extraction, and model inference—should be conducted to evaluate the total latency for real-time clinical deployment.

In summary, this paper advances the field of ECG-based HF classification by delivering a practical and scalable approach that effectively bridges sophisticated computational techniques with tangible clinical utility, particularly for underserved communities and point-of-care applications. To strengthen clinical interpretability, future research should incorporate explainable AI (XAI) techniques, including SHAP and LIME [39], to elucidate feature contributions and support clinical decision making.

## Figures and Tables

**Table 1:** Baseline clinical characteristics of the study cohort stratified by heart failure phenotype: HFpEF (n = 63), HFmrEF (n = 54), and HFrEF (n = 186).

Variable	Overall (n = 303)	HFpEF (n = 63)	HFmrEF (n = 54)	HFrEF (n = 186)
		LVEF ≥ 50%	LVEF 41–49%	LVEF ≤ 40%
LVEF (%)	25–82	50–82	41–49	25–40
Age (years)	18–89	18–79	40–89	40–89
Sex (Male)/(Female)	217/86	50/13	39/15	128/58
Diabetes (Yes)/(No)	114/189	26/37	18/36	70/116
Hypertension (Yes)/(No)	181/122	34/29	42/12	105/81

**Table 2:** Descriptions of ECG features extracted in this paper.

ECG Feature	Definition
RR_mean (ms)	Mean interval between successive R-peaks, reflecting average heart rate.
QRS_mean (ms)	Mean duration of the QRS complex, representing ventricular depolarization.
QT_mean (ms)	Average uncorrected QT interval between ventricular depolarization onset (QRS start) and repolarization (end of T wave).
P-wave Energy	Energy of the P wave, reflecting atrial depolarization.
QRS Energy	Energy of the QRS complex, reflecting ventricular contraction strength.
T-wave Energy	Energy of the T wave, indicating ventricular repolarization.

**Table 3:** Optimized hyperparameters for classification models.

Model	Tuned Hyperparameters	Optimal Setting
CatBoost	Iterations [500, 1000], Learning Rate [0.01, 0.1], Depth [6, 8, 10], Early Stopping Rounds [50, 100]	1000, 0.01, 8, 50
LightGBM	n_estimators [500, 1000], Learning Rate [0.01, 0.1], Max Depth [6, 8, 10], Num Leaves [15, 30]	1000, 0.01, 8, 30
XGBoost	n_estimators [500, 1000], Learning Rate [0.01, 0.1], Max Depth [6, 8, 10]	1000, 0.01, 8
Random Forest	n_estimators [100, 200, 300], Max Depth [5, 10, 20]	200, 10
Stacking	Base models optimized individually	Base models optimized individually

**Table 4:** ECG features [median (Q1–Q3)] for each HF phenotype.

ECG Feature	HFpEF (n = 63)	HFmrEF (n = 54)	HFrEF (n = 186)	*p*-Value *
RR_interval (ms)	905.9 (736.0–1075.8)	871.6 (697.1–1046.1)	855.7 (524.0–1187.4)	0.042
QRS_duration (ms)	123.4 (67.4–179.4)	136.7 (83.5–189.9)	143.0 (83.9–202.1)	0.008
QT_interval (ms)	343.9 (243.9–443.9)	371.3 (303.6–439.0)	347.2 (258.3–436.1)	0.156
P_Energy ^†^	0.8 (0.2–2.6)	0.7 (0.1–2.2)	1.4 (0.3–3.9)	0.023
QRS_Energy ^†^	0.8 (0.2–2.7)	0.6 (0.1–2.1)	0.9 (0.2–2.9)	0.187
T_Energy ^†^	0.7 (0.1–2.4)	0.6 (0.1–2.1)	1.3 (0.2–3.7)	0.031

* Kruskal–Wallis test for overall group differences. ^†^ Values are normalized; units depend on preprocessing (µV^2^·ms or arbitrary units).

**Table 5:** Performance comparison of classification models on HF phenotypes.

Model	Val Accuracy	Test Accuracy	Avg AUC	Avg F1	Training Time (s)
CatBoost	0.9804	0.9726	0.9965	0.9648	13.77
LightGBM	0.9866	0.9845	0.9989	0.9804	2.88
XGBoost	0.9824	0.9768	0.9973	0.9698	6.14
RandomForest	0.9654	0.9577	0.9963	0.9466	1.78
Stacking	0.9855	0.9819	0.9979	0.9768	121.37

**Table 6:** Comparison of recent studies on LVEF-based HF classification. Bold classifiers indicate the best performance (AGC: American, Greek cohorts).

Author (Year)	ECG Duration	Database	Method/Features	Classifier	Acc. (%)
Sona et al. [21]	1 h (24 h study)	AGC (229 patients)	TP, QT, QRS, ST, entropy, inst. freq.	TREE	91.2
				KNN	90.9
				SVM	87.8
				NN	84.3
Zhang et al. [26]	30 min	MIMIC-III (268); BIDMC; Fantasia; THEW	Spatial + temporal ECG features	CNN-LSTM	99.09
Alkhodari et al. [22]	NA	AGC (303 patient)	Clinical markers (BMI, diabetes, MI, VT, meds)	CNN	90.1
Kwon et al. [27]	10 s	34,103 patients (multi-hospital)	Demographics + QRS, T-wave, P-wave	CNN	86.6
**This paper **	1 min (20 min study)	MUSIC (303 patients)	RR, QT, QRS, P/T/QRS energy	**LightGBM **	**98.45 **
				CatBoost	97.26
				XGBoost	97.68
				RandomForest	95.77
				Stacking	98.19

## Data Availability

The ECG data used in this study are available from the MUSIC (Sudden Death in Heart Failure) database on PhysioNet at https://physionet.org/content/music-sudden-cardiac-death/1.0.1/, accessed on 25 August 2025. Derived features and analysis code are available from the corresponding author upon reasonable request.
